# Evaluation of different internal bleaching methods after application of two bioceramic formulations in revascularization treatment: an in vitro study

**DOI:** 10.1038/s41598-026-65462-z

**Published:** 2026-08-11

**Authors:** Reham Khaled Elghazawy, Dina Hamdy, Amr Medhat Eldeeb, Sarah Hossam Fahmy

**Affiliations:** 1 Pediatric Dentistry and Dental Public Health Department, Faculty of Dentistry, Ain Shams University, African Unity Organization Street, Cairo, Egypt; 2 Department of Conservative Dentistry, Faculty of Dentistry, Ain Shams University, African Unity Organization Street, Cairo, Egypt; 3 Department of Endodontics, Faculty of Dentistry, Ain Shams University, African Unity Organization Street, Cairo, Egypt

**Keywords:** Diseases, Health care, Medical research

## Abstract

To evaluate the staining potential of two bioceramic formulations, NeoPUTTY and NeoMTA Plus, in a revascularization model and to assess the bleaching efficacy of hydrogen peroxide with and without ultrasonic or diode laser activation at different application time intervals. Forty-two extracted permanent maxillary incisors were prepared for revascularization and randomly allocated to two groups: NeoPUTTY (GI) and NeoMTA Plus (GII). After 3 months, each group was further subdivided into three subgroups using the internal bleaching method: diode laser activation (a), ultrasonic activation (b), and no activation (c). CIEL*a*b* values were measured using the VITA Easyshade V spectrophotometer at pre-revascularization (T0), 3 months post-revascularization (T1), on the day of bleaching gel application (T2), and after one week (T3) and two weeks (T4) of bleaching gel application. All specimens showed significant decreases in L*, a*, and b* values at T1 (*p* < 0.001). Group I exhibited a non-significantly higher ΔE00 value between T0 and T1 than Group II (*p* = 0.142). All internal bleaching methods produced significant increases in L* and significant decreases in a* and b* at T3 compared with T1 (*p* < 0.001). GIa demonstrated a non-significantly higher ΔE00 value between T1 and T3 than GIb and GIc (*p* = 0.065), whereas GIIa exhibited a significantly higher ΔE00 value than GIIb and GIIc (*p* < 0.001). NeoPUTTY and NeoMTA Plus exhibited comparable staining potential. The bleaching efficacy of hydrogen peroxide was enhanced when the bleaching gel was activated with a diode laser and left in place for one week. A one-week application of a diode-laser-activated hydrogen peroxide bleaching gel may be an effective approach for reversing coronal discoloration following revascularization.

## Introduction

Revascularization has emerged as a viable alternative to apexification for the treatment of immature permanent teeth. The protocol primarily involves thorough disinfection of the root canal using intracanal medicaments, followed by the induction of an intraradicular blood clot. A barrier material is then placed over the clot to facilitate healing^1,2^. However, these procedures are inherently susceptible to coronal discoloration due to interactions among the tooth structure, bioceramic materials, intracanal medicaments, and blood pigments^3–5^.

Mineral trioxide aggregate (MTA) is frequently used in revascularization due to its excellent biocompatibility and sealing properties. However, it has several well-recognized limitations, including poor handling characteristics, a prolonged setting time, and a high potential for tooth discoloration^6^. Previous studies have shown that the metal oxide content of conventional MTA is primarily responsible for this discoloration. Consequently, the American Association of Endodontists (AAE) recommends the use of color-stable alternative materials during regenerative endodontic procedures^7^. Likewise, the European Society of Endodontology (ESE) recommends the use of bismuth oxide-free materials, particularly when they are placed coronal to the cementoenamel junction (CEJ)^1^.

NeoMTA Plus® (Avalon Biomed Inc., Bradenton, FL, USA) is a bioceramic material mixed with a water-based gel that uses tantalum oxide, rather than bismuth oxide, as its radiopacifier. It is specifically indicated for pulpotomy and regenerative endodontic procedures because of its color stability. Subsequently, the manufacturer introduced NeoPUTTY®, a premixed bioceramic putty formulation that enables immediate placement while reducing material waste, treatment costs, and chairside time, without compromising color stability. These characteristics make both materials particularly suitable for use in the esthetic zone, where even minor color changes may necessitate additional esthetic treatment^6^.

Despite the high prevalence of coronal discoloration following regenerative endodontic procedures, there is currently no consensus regarding the optimal management protocol. Internal bleaching remains the primary treatment option for discolored maxillary anterior teeth and can be performed using various oxidizing agents. These bleaching systems differ in their chemical composition, concentration, application duration, and activation methods, including heat, light, laser, and ultrasound^8,9^.

Among the available bleaching agents, hydrogen peroxide is widely recognized for its ability to penetrate enamel and dentin effectively. Its decomposition can be accelerated by heat, light, laser irradiation, or ultrasonic activation. Ultrasonic activation has been reported to increase dentin permeability^10^, facilitating deeper penetration of the bleaching agent into the tooth structure and thereby enhancing bleaching efficacy^11^. Likewise, diode-laser activation accelerates the decomposition of hydrogen peroxide, generating reactive oxygen species that can yield faster and/or greater color improvement. This effect has been demonstrated with several diode laser wavelengths, including 445 nm, 810–980 nm, and 940 nm^12^. Furthermore, laser-assisted internal bleaching has achieved whitening outcomes comparable to those of multi-day walking bleach protocols while substantially reducing clinical treatment time and maintaining intrapulpal temperatures within safe limits when appropriate irradiation parameters are used^13^. Collectively, the enhanced diffusion achieved through ultrasonic activation and the increased generation of reactive radicals induced by diode laser photoactivation provide a biologically plausible and experimentally supported rationale for improving the efficiency and effectiveness of internal bleaching^10–12^.

Therefore, the primary objective of this study was to evaluate the staining potential of NeoPUTTY and NeoMTA Plus in the presence of an intraradicular blood clot using an in vitro revascularization model. The secondary objective was to evaluate the bleaching efficacy of hydrogen peroxide, with and without ultrasonic or diode-laser activation, at varying application durations for reversing coronal discoloration following revascularization.

The null hypotheses were as follows: (1) the revascularization protocols evaluated would not induce clinically detectable color changes, and (2) no significant differences would exist in the bleaching efficacy among the tested internal bleaching methods.

## Materials and methods

### Ethical approval

The study protocol was reviewed and approved by the Research Ethics Committee of the Faculty of Dentistry, Ain Shams University (Approval No. FDASU-Rec ER012435). The study was conducted in accordance with the principles of the Declaration of Helsinki.

### Sample size calculation

A priori power analysis was performed to ensure adequate statistical power to reject the null hypothesis of no difference in color change among the study groups. Assuming an alpha (α) level of 0.05, a statistical power of 95% (β = 0.05), and an effect size (f) of 0.792 derived from a previous study^14^, the minimum required sample size was calculated to be 42 specimens (21 per group and 7 per subgroup). Sample size calculations were performed using G*Power (version 3.1.9.7).

### Specimen preparation

Maxillary central and lateral incisors extracted for periodontal reasons, with no caries, cracks, previous endodontic treatment, or coronal restorations, were collected for this study. The teeth were stored in physiological saline until use. Calculus deposits were removed using periodontal curettes. The apical portions of the roots were sectioned with a water-cooled diamond disc to obtain standardized root segments measuring 8 mm in length. Root canals were enlarged sequentially using #1–#5 Peeso reamers (Mani Inc., Tochigi, Japan), followed by irrigation with 17% EDTA (Imicryl, Konya, Turkey) and 2.5% sodium hypochlorite (NaOCl) (Caglayan Kimya, Konya, Turkey) for 5 min each. Finally, the apical openings were sealed with composite resin^15,16^.

### Intracanal medication placement

Following access cavity preparation, calcium hydroxide was placed into the enlarged root canals using a Lentulo spiral. A cotton pellet was positioned within the access cavity, which was then sealed with Cavit (3M ESPE, St. Paul, MN, USA). The specimens were incubated at 37 °C under 100% humidity for two weeks. After the incubation period, the temporary restoration was removed, and the canals were irrigated with 5 mL of saline to eliminate the calcium hydroxide.

### Blood insertion and coronal sealing

Fresh human blood was collected from a volunteer member of the research team after obtaining informed consent, then injected into the root canals. Collacote (Integra LifeSciences Corporation, Princeton, NJ, USA) was cut into small sections and placed into each canal to a level 3 mm below the CEJ.

The teeth were then randomly allocated into two groups (*n* = 21 per group):

- Group I (GI): NeoPUTTY
- Group II (GII): NeoMTA Plus

For both groups, the assigned bioceramic material was placed over the blood-soaked Collacote. A moist cotton pellet was placed over the material, and the access cavity was temporarily sealed with glass ionomer cement (3M ESPE, St. Paul, MN, USA). The specimens were incubated at 37 °C under 100% humidity for 12 weeks. After incubation, the temporary restorations were removed, and tooth color was recorded (T1).

### Bleaching protocols and activation methods

A 40% hydrogen peroxide bleaching gel (POWER WHITENING, Whitesmile, Germany) was applied to all specimens in a standardized 1–2 mm layer, consistent with established internal bleaching protocols that use this thickness to regulate heat transfer during light activation^10–13^.

### Diode-laser activation

A 940-nm diode laser was applied for short, repeated 30-s cycles, as validated in-office bleaching parameters. The laser handpiece was maintained in a non-contact position above the bleaching gel surface, as previously described for diode laser-assisted bleaching^13^.

### Ultrasonic activation

Ultrasonic activation was performed using a 28-kHz ultrasonic device. The ultrasonic tip was placed in light contact with, or slightly immersed in, the bleaching gel to facilitate effective transmission of acoustic energy. Activation was performed for 60 s, consistent with peroxide activation intervals reported for sonochemical bleaching systems^17^.

Each experimental group was randomly subdivided into three subgroups (*n* = 7 per subgroup) according to the bleaching protocol.

**Group I (GI): NeoPUTTY**

*GIa:* Hydrogen peroxide gel activated with a 940-nm diode laser (EpicX, Biolase, USA) for 30 s at 2 W, 40 Hz, and 50 mJ.

*GIb:* Hydrogen peroxide gel activated with an ultrasonic device (NSK, Kanuma, Tochigi, Japan) for 60 s at 28 kHz.

*GIc:* Hydrogen peroxide gel without activation.

**Group II (GII): NeoMTA plus**

*GIIa:* Hydrogen peroxide gel activated with a 940-nm diode laser (EpicX, Biolase, USA) for 30 s at 2 W, 40 Hz, and 50 mJ.

*GIIb:* Hydrogen peroxide gel activated with an ultrasonic device (NSK, Kanuma, Tochigi, Japan) for 60 s at 28 kHz.

*GIIc:* Hydrogen peroxide gel without activation.

### Tooth color evaluation

Tooth color was measured using a digital spectrophotometer (VITA Easyshade V; Vita Zahnfabrik, Bad Säckingen, Germany). Measurements were obtained on the labial surface at a standardized point located 3 mm above the center of the CEJ under consistent laboratory lighting conditions by a single calibrated operator. Before each measurement, the spectrophotometer was calibrated using the manufacturer’s calibration port.

Color measurements were recorded according to the CIE L*a*b* color system (Commission Internationale de l’Éclairage, Vienna, Austria). The L* coordinate represents lightness, ranging from 0 (black) to 100 (white). The a* coordinate represents the red-green axis, with positive values indicating redness and negative values indicating greenness. The b* coordinate represents the yellow-blue axis, with positive values indicating yellowness and negative values indicating blueness^18^.

Tooth color was evaluated at baseline before revascularization (T0), three months after revascularization (T1), on the day of bleaching gel application (T2), one week after bleaching (T3), and two weeks after bleaching (T4).

To determine color differences between the baseline and every measurement point, ΔE_00_ values were calculated using the following Eq. (19):

$$\Delta {\mathrm{E}}_{00} = \left[ {\left( {\Delta {\mathrm{L}}\prime /{\mathrm{k}}_{{\mathrm{L}}} {\mathrm{S}}_{{\mathrm{L}}} } \right)^{2} + \left( {\Delta {\mathrm{C}}\prime /{\mathrm{k}}_{{\mathrm{c}}} {\mathrm{S}}_{{\mathrm{c}}} } \right)^{2} + \left( {\Delta {\mathrm{H}}\prime /{\mathrm{k}}_{{\mathrm{H}}} {\mathrm{S}}_{{\mathrm{H}}} } \right)^{2} + {\mathrm{R}}_{{\mathrm{T}}} \left( {\Delta {\mathrm{C}}\prime /{\mathrm{k}}_{{\mathrm{c}}} {\mathrm{S}}_{{\mathrm{c}}} } \right)\left( {\Delta {\mathrm{Hv}}\prime /{\mathrm{k}}_{{\mathrm{H}}} {\mathrm{S}}_{{\mathrm{H}}} } \right)} \right] ^{\raise.5ex\hbox{$\scriptstyle 1$}\kern-.1em/ \kern-.15em\lower.25ex\hbox{$\scriptstyle 2$} }$$

Where: An excellent match occurs at ΔE_00_ ≤ 0.8, Acceptable match occurs at ΔE_00_ > 0.8, ≤ 1.8, Moderately unacceptable mismatch (mismatch type a) occurs at ΔE_00_ > 1.8, ≤ 3.6, Clearly unacceptable mismatch (mismatch type b) occurs at ΔE_00_ > 3.6, ≤ 5.4, Extremely unacceptable mismatch (mismatch type c) occurs at ΔE_00_ > 5.4.

### Statistical analysis

Continuous variables were expressed as mean ± standard deviation (SD). Data normality was assessed by visual inspection of the distribution and confirmed using the Shapiro–Wilk test. Because the data were normally distributed, linear mixed-effects models were constructed to evaluate the effects of the experimental variables on the color parameters. Model assumptions, including linearity, were assessed by visual inspection of residual-versus-fitted plots, while the normality of both fixed- and random-effect residuals was evaluated using the methods described above. Following model fitting, estimated marginal means were compared using Welch’s F-tests with the Kenward–Roger approximation for degrees of freedom. P-values were adjusted for multiple comparisons using Holm’s correction. Effect sizes were calculated and interpreted according to Cohen^19^. Statistical significance was established at *p* < 0.05. All statistical analyses were performed using R statistical software (version 4.5.2; R Core Team, 2025, *R: A Language and Environment for Statistical Computing*; R Foundation for Statistical Computing, Vienna, Austria; https://www.R-project.org/).

## Results

All specimens displayed notable discoloration (ΔE_00_ > 5.4) at T1. The NeoPUTTY group showed slightly higher ΔE_00_ values, with no significant difference from the NeoMTA Plus group (Table 1).

**Table 1:** Effect of bioceramic formulation on tooth discoloration.

ΔE_00_ (T0-T1) (Mean ± SD)		*p*-value	PES (95% CI)
NeoPUTTY (GI)	NeoMTA Plus (GII)		
9.57 ± 2.55	8.46 ± 2.48	0.142	0.01 (0.00–0.07)

PES, partial eta squared; CI, confidence interval.

### Bleaching effect of different internal bleaching methods

All internal bleaching methods were effective at producing a perceptible color change relative to the discolored state (ΔE00 > 1.8). Still, the degree of color change depended on both the internal bleaching method and the application interval. GIa showed non-significantly higher ΔE_00_ between both T1-T3 and T1-T4 than GIb and GIc (*p* = 0.065), while GIIa resulted in significantly higher ΔE_00_ between both T1-T3 and T1-T4 compared to GIIb and GIIc (*p* < 0.001). Time-dependent color change within each subgroup showed that the ΔE_00_ values between T1-T3 were significantly higher than those between T1-T2 for all subgroups (*p* ˂ 0.05) except for GIc and GIIc, in which the ΔE_00_ values were not significantly different. the ΔE_00_ values between T1-T4 were non-significantly different than those between T1-T3 for all subgroups (*p* > 0.05) **(**Table 2**)**.

**Table 2:** Bleaching efficacy of different internal bleaching methods relative to the discolored state.

Group	Interval	ΔE_00_ (Mean ± SD)			*p*-value	PES (95% CI)
		Bleaching + Laser activation (a)	Bleaching + Ultrasonic activation (b)	Bleaching only (c)		
NeoPUTTY (GI)	T1-T2	7.78 ± 1.34^Ab^	4.52 ± 1.04^Bb^	6.79 ± 4.06^ABb^	0.040*	0.04 (0.00–0.11)
	T1-T3	12.20 ± 3.24^Aa^	9.41 ± 1.87^Aa^	9.70 ± 4.47^Aab^	0.065ns	0.04 (0.00–0.10)
	T1-T4	12.80 ± 3.30^Aa^	10.29 ± 1.80^Aa^	10.77 ± 4.13^Aa^	0.125ns	0.03 (0.00–0.09)
	*p*-value	< 0.001*	< 0.001*	< 0.001*		
	PES (95% CI)	**0.11 (0.04–0.19)**	**0.14 (0.06–0.22)**	**0.07 (0.01–0.13)**		
NeoMTA Plus (GII)	T1-T2	6.26 ± 2.20^Ab^	5.66 ± 3.07^ABb^	3.07 ± 0.95^Ba^	0.036*	0.04 (0.00–0.12)
	T1-T3	11.20 ± 3.88^Aa^	8.59 ± 2.78^Ba^	4.75 ± 1.22^Ca^	< 0.001*	0.14 (0.05–0.24)
	T1-T4	12.03 ± 2.43^Aa^	7.10 ± 2.82^Bab^	5.52 ± 1.19^Ba^	< 0.001*	0.15 (0.06–0.25)
	*p*-value	< 0.001*	0.023*	**0.063**		
	PES (95% CI)	**0.14 (0.06–0.22)**	**0.03 (0.00–0.09)**	**0.03 (0.00–0.07)**		

PES, partial eta squared; CI, confidence interval. Values with different upper and lowercase superscripts within the same horizontal row and vertical column, respectively, are significantly different; *significant (*p* < 0.05). Significant values are in bold.

L* values showed a significant decrease at T1 compared to T0 across all subgroups (*p *˂ 0.001). This was followed by a significant increase at T2 and T3 (*p* < 0.001). However, no significant change was observed at T4 compared with T3 across all subgroups (*p* > 0.05), except for GIIb (Table 3). All groups exhibited positive a* and b* values at T0, indicating reddish and yellowish components, respectively. A progressive reduction in values was observed over time across all subgroups, indicating a shift towards greenness (Table 3). Also, a progressive reduction in b* values was observed over time across all subgroups, indicating a shift towards blueness (Table 3).

**Table 3:** CIE L*a*b* color coordinates measured at different time intervals.

Color coordinate	Group	Subgroup	(Mean ± SD)					*p*-value	PES (95% CI)
			T0	T1	T2	T3	T4		
L*	GI	GIa	84.36 ± 3.90^B^	71.67 ± 1.25^C^	82.29 ± 1.32^B^	88.00 ± 3.53^A^	88.55 ± 4.34^A^	< 0.001*	0.56 (0.45–0.64)
		GIb	83.93 ± 3.41^BC^	75.26 ± 3.51^D^	80.71 ± 4.39^C^	87.09 ± 4.66^AB^	87.81 ± 4.99^A^	< 0.001*	0.42 (0.29–0.51)
		GIc	80.38 ± 4.94^AB^	70.34 ± 7.23^C^	78.62 ± 5.15^B^	82.57 ± 6.53^A^	83.26 ± 5.89^A^	< 0.001*	0.42 (0.29–0.52)
	GII	GIIa	80.39 ± 3.68^B^	74.77 ± 3.18^C^	82.65 ± 2.41^B^	88.84 ± 2.23^A^	87.73 ± 2.99^A^	< 0.001*	0.47 (0.35–0.56)
		GIIb	87.03 ± 1.88^AB^	78.21 ± 5.59^C^	85.24 ± 2.12^B^	89.71 ± 3.16^A^	85.78 ± 6.30^B^	< 0.001*	0.33 (0.20–0.43)
		GIIc	82.92 ± 1.91^A^	76.33 ± 3.90^B^	79.26 ± 2.81^AB^	81.59 ± 2.30^A^	80.20 ± 3.41^AB^	< 0.001*	0.14 (0.04–0.24)
a*	GI	GIa	5.21 ± 0.85^A^	1.11 ± 2.03^B^	− 0.17 ± 1.50^C^	− 2.46 ± 0.73^D^	− 3.12 ± 0.36^D^	< 0.001*	0.69 (0.61–0.75)
		GIb	5.20 ± 1.62^A^	1.71 ± 1.97^B^	0.12 ± 0.92^C^	− 2.28 ± 0.64^D^	− 2.72 ± 1.24^D^	< 0.001*	0.68 (0.59–0.74)
		GIc	7.16 ± 1.21^A^	2.43 ± 1.84^B^	1.12 ± 1.02^C^	− 0.71 ± 0.73^D^	− 1.52 ± 0.93^D^	< 0.001*	0.70 (0.62–0.76)
	GII	GIIa	5.86 ± 1.66^A^	0.51 ± 2.68^B^	− 1.10 ± 1.11^C^	− 2.05 ± 0.50^CD^	− 3.03 ± 0.67^D^	< 0.001*	0.71 (0.64–0.77)
		GIIb	6.14 ± 2.53^A^	0.26 ± 1.65^B^	− 0.58 ± 1.66^BC^	− 2.22 ± 1.29^D^	− 1.54 ± 2.41^CD^	< 0.001*	0.69 (0.61–0.75)
		GIIc	7.52 ± 2.17^A^	0.84 ± 1.67^B^	1.15 ± 1.50^B^	− 0.44 ± 1.22^C^	− 1.30 ± 1.51^C^	< 0.001*	0.71 (0.63–0.76)
b*	GI	GIa	45.62 ± 3.29^A^	29.99 ± 2.25^B^	32.19 ± 2.78^B^	24.07 ± 3.09^C^	23.47 ± 5.57^C^	< 0.001*	0.56 (0.45–0.64)
		GIb	46.44 ± 6.56^A^	34.64 ± 3.49^B^	31.04 ± 2.52^BC^	26.64 ± 3.49^CD^	24.83 ± 2.13^D^	< 0.001*	0.54 (0.43–0.62)
		GIc	47.50 ± 4.64^A^	37.19 ± 5.46^B^	32.75 ± 4.71^BC^	31.15 ± 7.08^C^	29.07 ± 5.50^C^	< 0.001*	0.46 (0.34–0.55)
	GII	GIIa	44.60 ± 3.83^A^	32.74 ± 5.66^B^	28.21 ± 4.83^C^	23.99 ± 4.30^D^	18.39 ± 4.70^E^	< 0.001*	0.61 (0.51–0.68)
		GIIb	47.87 ± 3.43^A^	33.09 ± 3.01^B^	33.20 ± 2.42^B^	27.29 ± 2.31^C^	25.26 ± 5.21^C^	< 0.001*	0.55 (0.44–0.63)
		GIIc	51.70 ± 5.44^A^	35.48 ± 7.42^B^	33.71 ± 6.73^B^	32.10 ± 4.70^BC^	28.10 ± 5.49^C^	< 0.001*	0.57 (0.46–0.64)

PES, partial eta squared; CI, confidence interval. Values with different superscripts within the same horizontal row are significantly different; *significant (*p* < 0.05).

L* values showed a significant decrease at T1 compared to T0 across all subgroups (*p* ˂ 0.001). This was followed by a significant increase at T2 and T3 (*p* ˂ 0.001). However, no significant change was observed at T4 compared with T3 across all subgroups (*p* > 0.05), except for GIIb (Table 3). All groups exhibited positive a* and b* values at T0, indicating reddish and yellowish components, respectively. A progressive reduction in values was observed over time across all subgroups, indicating a shift towards greenness (Table 3). Also, a progressive reduction in b* values was observed over time across all subgroups, indicating a shift towards blueness (Table 3).

Although some calcium silicate cements may initially appear to preserve dental esthetics, substantial evidence indicates that these materials can induce tooth discoloration over time, particularly in the presence of blood, as occurs during revascularization procedures. Nevertheless, the precise mechanisms underlying this long-term discoloration remain incompletely understood. Therefore, the present study compared the discoloration potential of two bioceramic formulations, NeoPUTTY and NeoMTA Plus, which were expected to induce only minimal color changes, and evaluated the reversibility of the resulting discoloration using hydrogen peroxide-based internal bleaching with or without ultrasonic or diode laser activation. The CIEDE2000 (ΔE_00_) was applied to calculate ΔE_00_ because it has been shown to agree with human visual assessments of color difference at 95%, providing a better fit than CIELab (ΔE*_ab_), which has only 75% agreement. Paravina et al.^20^, suggested that excellent match occurs at perceptibility threshold (PT) ≤ 0.8 ΔE_00_ units, acceptable match occurs at acceptability threshold (AT) ≤ 1.8 ΔE_00_ units but > 0.8 ΔE_00_ units, moderately unacceptable mismatch (mismatch type a) occurs at AT ≤ 3.6 ΔE_00_ units but > 1.8 ΔE_00_ units, clearly unacceptable mismatch (mismatch type b) occurs at AT ≤ 5.4 ΔE_00_ units but > 3.6 ΔE_00_ units and extremely unacceptable mismatch (mismatch type c) occurs at AT > 5.4 ΔE_00_ units.

The findings of the present study led to the rejection of both null hypotheses. After three months of the revascularization protocol, both groups exhibited significant tooth discoloration. Furthermore, significant differences in bleaching efficacy were observed among the tested internal bleaching methods. (ΔE_00_ > 5.4 units), characterized by decreased L* values (darkening), a* values (shift toward green), and b* values (shift toward blue). The reduction in a* and b* values could be related to the penetration of erythrocytes into dentin and the accumulation of hematin molecules upon hemolysis into the dentinal tubules^1,21^. Although the difference was not statistically significant, the NeoPUTTY group exhibited slightly greater discoloration than the NeoMTA Plus group. This finding may be attributed to the incorporation of blood into the incompletely set putty, whereby the material’s initial porosity facilitates the absorption of erythrocytes^22,23^.

Blood contamination has been shown to significantly increase the discoloration of bioceramic materials, including those with inherently good color stability^6,24,25^. During redox reactions, ferrous (Fe^2^⁺) ions in hemoglobin are oxidized to ferric (Fe^3^⁺) ions, resulting in the formation of darker pigments that contribute to tooth staining. Lenherr et al. and Žižka et al. suggested that allowing bioceramic materials to set before exposure to blood may reduce discoloration^26,27^. Supporting this hypothesis, Nazzal et al.^28^ reported clinically relevant post-revascularization discoloration (ΔE = 7.9) even when coronal plug materials free of bismuth oxide were used. Conversely, in the absence of blood contamination, NeoPUTTY maintained excellent color stability for up to 12 months^29^. Collectively, these findings suggest that discoloration following regenerative endodontic procedures is multifactorial and cannot be attributed solely to the radiopacifier or the intracanal medicament.

The reduction in L* values observed after revascularization may be attributed to bioceramic-mediated tooth discoloration. The proposed mechanisms primarily involve radiopacifier oxidation, chemical interactions between the bioceramic material and dentin, and environmental factors such as NaOCl and blood contamination. Comparative studies have demonstrated that although NeoMTA Plus exhibits improved physical properties compared with conventional MTA, it remains susceptible to clinically perceptible discoloration. Moreover, NeoPUTTY has been reported to exhibit greater intratubular penetration, which may increase the interaction between its constituents and dentin and thereby contribute to discoloration^23,24^. In contrast to the present findings, Keskin et al.^30^ reported greater discoloration with NeoMTA Plus than with NeoPUTTY and Biodentine.

Internal bleaching induced multidimensional color changes, shifting the overall tooth color toward a lighter and less saturated appearance. The marked increase in L* values reflected enhanced lightness, whereas the progressive reduction in a* values, reaching negative values after one to two weeks, indicated a shift toward the green axis. Similarly, the decrease in b* values indicated a shift toward the blue end of the color space, reflecting a reduction in the reddish-yellow hue of the tooth structure^8^. These color changes were most pronounced in the diode laser-activated groups. In contrast, one previous study reported no significant difference in bleaching efficacy between diode-laser-activated and non-activated hydrogen peroxide^31^.

High-concentration hydrogen peroxide (35%–40%) is effective for tooth whitening; however, it may also cause adverse effects, including tooth sensitivity, pulpal irritation, alterations in enamel surface texture, increased surface roughness, and reduced microhardness^8,9,14,31^. To minimize these undesirable effects, laser-activated bleaching techniques have been developed, enabling comparable whitening outcomes while using approximately 24% less hydrogen peroxide than conventional protocols^32,33^. This approach is based on a thermochemical mechanism in which elevated temperatures accelerate the decomposition of hydrogen peroxide into highly reactive oxygen radicals that oxidize chromogenic compounds, thereby shortening treatment time^32,33^. Laser-assisted bleaching has consistently demonstrated superior performance over conventional bleaching for resistant discoloration. Specifically, diode and Nd:YAG lasers have been shown to reduce treatment time^31^, potassium titanyl phosphate (KTP) lasers have achieved greater color improvement^34^, and photon-induced photoacoustic streaming (PIPS) combined with 35% hydrogen peroxide has produced superior immediate and sustained whitening outcomes^4^. In contrast, some light-activated bleaching systems have been reported to accelerate the initial whitening response without providing a significant long-term advantage over conventional techniques after multiple treatment sessions^31^.

Ultrasonic activation produced comparatively modest color improvement in the present study. Similarly, previous studies have reported that ultrasonic activation of hydrogen peroxide or carbamide peroxide does not significantly enhance bleaching efficacy, as measured by color change (ΔE*) or lightness (ΔL*), compared with conventional internal bleaching techniques^11^, despite its reported ability to increase dentin permeability^35^. This finding may be explained by insufficient vibration intensity or inadequate activation time. In the present study, the ultrasonic device was operated at a relatively low frequency (28 kHz) to minimize heat generation and reduce the risk of iatrogenic damage while still providing sufficient energy to activate the bleaching agent. Therefore, further studies investigating different ultrasonic frequencies, power settings, and application durations are warranted to optimize the efficacy of ultrasonic-assisted internal bleaching.

The greatest bleaching response was observed during the first week of treatment, whereas only marginal and non-significant changes occurred during the second week. This finding suggests progressive depletion of the active components of the bleaching agent over time. Previous studies have similarly demonstrated that the greatest color improvement occurs immediately after treatment or within the first week, followed by diminishing returns in subsequent weeks^36–38^. For example, Matis et al.^36^ reported a 51% reduction in color change (ΔE) by the second week, indicating a plateau in bleaching efficacy. Likewise, Darriba et al.^39^ found that reapplying the bleaching agent after one week produced greater whitening than simply extending the application time without reapplication. Similarly, two sessions of laser-assisted bleaching performed at a one-week interval achieved more persistent color improvement than a single treatment session^8,33^. In addition, studies evaluating the stability and storage of bleaching agents have shown that their efficacy declines over time as the active peroxide components become depleted^33,40^. Collectively, these findings underscore the importance of informing patients about the diminishing benefits of prolonged bleaching and the potential advantages of reapplying the bleaching agent when additional whitening is desired^38,40–42^.

The present study has several limitations. As an in vitro investigation, its findings cannot be directly extrapolated to the clinical setting, where the oral environment is influenced by numerous biological and environmental factors, including saliva, dietary habits, temperature fluctuations, masticatory forces, and interindividual variability. Furthermore, important knowledge gaps remain regarding the long-term clinical performance of contemporary bioceramic materials and the molecular mechanisms underlying tooth discoloration following regenerative endodontic procedures. Future research should therefore focus on well-designed long-term clinical trials, mechanistic investigations, and the development of standardized protocols for the assessment, prevention, and management of post-revascularization discoloration.

## Conclusion and future recommendations

Within the limitations of this in vitro study, NeoPUTTY and NeoMTA Plus induced comparable levels of tooth discoloration following regenerative endodontic treatment (RET). Internal bleaching effectively reduced discoloration regardless of the activation method; however, diode-laser-activated bleaching demonstrated superior efficacy compared with ultrasonic activation and hydrogen peroxide gel alone. Furthermore, the laser-activated bleaching protocol achieved its greatest whitening effect after one week of application, with no significant additional improvement after two weeks.

These findings support the use of diode laser-activated internal bleaching as an effective approach for managing coronal discoloration following RET. Future studies should validate these findings through long-term randomized clinical trials, investigate the molecular mechanisms underlying discoloration and bleaching, and optimize laser and ultrasonic activation parameters to establish evidence-based clinical protocols.

## Data Availability

The raw data derived from the VITA easy shade readings for the color coordinates are available, but restrictions apply to the availability of these data, which were used under licence for the current study and so are not publicly available. The data are, however, accessible upon request and with the permission of researcher support center at the faculty of Dentistry, at Ain shams university.
